# Local Control Failure After Five-Fraction Stereotactic Radiosurgery Alone for Symptomatic Brain Metastasis From Squamous Cell Lung Carcinoma Despite 43 Gy to Gross Tumor Margin With Internal Steep Dose Increase and Tumor Shrinkage During Irradiation

**DOI:** 10.7759/cureus.38645

**Published:** 2023-05-06

**Authors:** Kazuhiro Ohtakara, Kuniaki Tanahashi, Takeshi Kamomae, Eiji Ito, Kojiro Suzuki

**Affiliations:** 1 Department of Radiation Oncology, Kainan Hospital Aichi Prefectural Welfare Federation of Agricultural Cooperatives, Yatomi, JPN; 2 Department of Radiology, Aichi Medical University, Nagakute, JPN; 3 Department of Neurosurgery, Gifu Prefectural Tajimi Hospital, Tajimi, JPN; 4 Department of Neurosurgery, Nagoya University Graduate School of Medicine, Nagoya, JPN; 5 Radioisotope Research Center, Nagoya University, Nagoya, JPN; 6 Department of Radiology, Nagoya University Graduate School of Medicine, Nagoya, JPN

**Keywords:** nintedanib, neuroendoscopy, squamous cell carcinoma, lung cancer, linear-quadratic model, biological effective dose, five fraction, stereotactic radiosurgery, fractionation, brain metastasis

## Abstract

Five-fraction (fr) stereotactic radiosurgery (SRS) is increasingly being applied to large brain metastases (BMs) >2-3 cm in diameter, for which 30-35 Gy is the commonly prescribed dose. Since 2018, to further enhance both safety and efficacy, we have limited the five-fr SRS to approximately ≤3 cm BMs and adopted our own modified dose prescription and distribution: 43 and 31 Gy cover the boundaries of the gross tumor volume (GTV) and 2 mm outside the GTV, respectively, along with a steep dose increase inside the GTV boundary, that is, an intentionally very inhomogeneous GTV dose. Herein, we describe a case of symptomatic BM treated with five-fr SRS using the above policy, which resulted in a maximum tumor response with nearly complete remission (nCR) followed by gradual tumor regrowth despite obvious tumor shrinkage during irradiation.

A 71-year-old man who had previously undergone surgery for squamous cell carcinoma (SCC) of the lungs presented with right-sided hemiparesis attributed to the para-falcine BM (27 mm in maximum diameter, 5.38 cm^3^). The BM was treated with five-fr SRS, with 99.2% of the GTV covered with 43 Gy and 59% isodose. Neurological symptoms improved during SRS, and obvious tumor shrinkage and mitigation of perilesional edema were observed upon completion of SRS. No subsequent anti-cancer pharmacotherapy was administered due to idiopathic pulmonary fibrosis (IPF). Despite a maximum response with nCR at four months, the tiny residual enhancing lesion gradually enlarged from 7.7 months to 22.7 months without neurological worsening. Although a consistent T1/T2 mismatch suggested the dominance of brain radionecrosis, ^11^C-methionine positron emission tomography showed increased uptake in the enhancing lesion. Pathological examination after total lesionectomy at 24.6 months revealed viable tumor tissue. Post-SRS administration of nintedanib for IPF may have provided some anti-tumor efficacy for lung SCC and may mitigate the adverse effects of SRS. The present case suggests that even ≥43 Gy with ≤60% isodose to the GTV boundary and ≥31-35 Gy to the 2 mm outside the GTV are insufficient to achieve long-term local tumor control by five-fr SRS alone in some large BM from lung SCC.

## Introduction

Stereotactic radiosurgery (SRS), with appropriate design and planning, is a valuable local treatment option for non-disseminated and non-miliary brain metastases (BMs) [1]. Certain anti-cancer drugs can modify the efficacy and toxicity of SRS [2,3]. In particular, long-term local control and safety are expected for SRS in patients with isolated and limited BMs without extracranial active disease who do not receive anti-cancer pharmacotherapy [4,5]. In such cases, the success or failure of SRS can have a significant impact on the patient’s clinical course, and the true value of SRS will be tested [4]. Local curability beyond temporary symptom palliation is required in such clinical scenarios [2,4,5]. 

In single-fraction (sf) SRS, a marginal dose of ≥24 Gy provides approximately 95% one-year local tumor control [1], whereas the risk of symptomatic brain radionecrosis (SBR) is significantly increased when the volume of 12 Gy or more irradiated to the surrounding brain (V_12 Gy_) exceeds 5 cm^3^ [5-7]. The BMs to which sfSRS can be effectively and safely applied are limited to approximately ≤15 mm in diameter [5,7]. Thus, optimal targets are more limited than conventional perceptions [5]. Recently, multi-fraction (mf) SRS, such as five-fraction (fr), has been frequently applied to BMs that are not amenable to sfSRS [1,3,4,7]. Prescribed doses of 30-35 Gy to margin-added planning target volumes (PTVs) are commonly used; however, the one-year local control rate remains at approximately 80% [1,7]. In addition, the adverse radiation effect (ARE) significantly increases when the volume irradiated to ≥24 Gy, including the lesion (24 Gy volume), exceeds 20 cm^3^ [6,7]. Since 2009, we have applied five-fr SRS to approximately 2-3.5 cm BMs [8]. Although safety had improved, including a significant reduction in the SBR, there was room for improvement in local control.

Kogo et al. reported excellent local control of BMs from non-small cell lung cancer (NSCLC) at a biologically effective dose (BED) of ≥80 Gy in 2-10 fr, median 3, SRS [9]. The BED was defined based on the linear-quadratic (LQ) model with an alpha/beta ratio of 10 (BED_10_) [9,10]. Although conventionally prescribed doses are frequently compromised for larger tumors, they emphasize ensuring a BED_10_ of 80 Gy or more, regardless of the tumor volume [4,9]. The PTV was defined as the gross tumor volume (GTV) + 1-2 mm. They covered 95% of the PTV by a BED_10_ of ≥80 Gy with 90% isodose surface (IDS) relative to the maximum dose (D_max_), homogeneous GTV dose. The 1- and 1.5-year local control rates were 85% and <75%, respectively, for tumors >2 cm in size, where there is still room for improvement [9]. Several cases of SBR refractory to steroids were reported [9]. Thereafter, there are likely no other facilities that treat with a BED_10_ of ≥80 Gy.

Since 2018, to improve both safety and efficacy, we have limited the target BM of 5-fr SRS to approximately ≤3 cm and adopted our own modified method for dose prescription and distribution: optimizing the dose gradient inside and outside the GTV boundary. Specifically, 43 and 31 Gy (BED_10_ 80 Gy and 50 Gy) covered the boundaries of the GTV and 2 mm outside the GTV, respectively, along with a steep dose increase inside the GTV boundary, that is, a very inhomogeneous GTV dose [3,4,10-12]. Herein, we describe a case of symptomatic BM treated with five-fr SRS alone using the above policy, which resulted in a maximum tumor response with nearly complete remission (nCR) followed by gradual tumor regrowth despite obvious tumor shrinkage during irradiation.

A part of this study was previously presented at the 30th Annual Meeting of the Japanese Society for Stereotactic Radiosurgery, held online from June 11 to July 8, 2021.

## Case presentation

**Figure 1:**
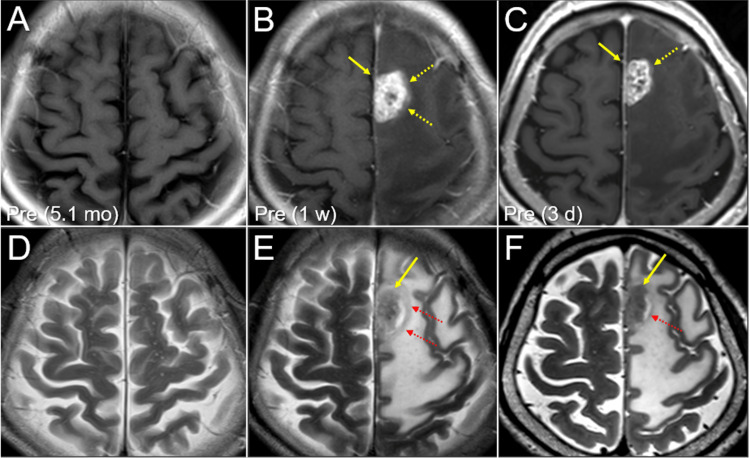
Magnetic resonance images before five-fraction stereotactic radiosurgery The images show contrast-enhanced (CE) axial T1-weighted images (WI) (A-C); axial T2-WI (D-F); 1.5-Tesla images for conventional diagnosis (A, B, D, and E); 3-Tesla images for treatment planning (C, F); 5.1 months (mo) (A, D), one week (w) (B, E), and three days (d) (C, F) before stereotactic radiosurgery (SRS). (A–F): All images are shown at the same magnification and coordinates under co-registration and fusion. (A, D): The normal morphology of the left superior and median frontal gyri can be seen before the development of brain metastasis (BM). (B, C, E, F): A heterogeneously enhanced mass lesion (arrows in B and C) is observed in the left frontal lobe, with broad attachment to the falcine dura, concomitant with extensive perilesional edema involving the primary and supplementary motor areas. (E, F): An irregularly-shaped mass lesion (arrows in E and F) with slightly high heterogeneous intensity is surrounded by a single-layered structure (dashed arrows in E and F) with slightly high intensity. A high-intensity component intervenes between them. The faint contrast exudation (dashed arrows in B and C) spreading outside the visible mass on T2-WI does not extend to the layered structure on T2-WI.

A 71-year-old right-handed man presented with right-sided hemiparesis and walking difficulty. Nine months earlier, the patient underwent a left lower lobectomy with regional lymph node dissection for squamous cell carcinoma (SCC) of the lungs. Before surgery, the patient was clinically diagnosed with idiopathic pulmonary fibrosis (IPF), which was pathologically verified at the time of surgery. Pirfenidone was administered perioperatively to prevent the acute exacerbation (AE) of IPF. Adjuvant anti-cancer medications were avoided due to IPF. Magnetic resonance imaging (MRI) revealed that the hemiparesis was attributed to para-falcine BM (27 mm in diameter, 5.38 cm3) in the left superior and medial frontal gyri (Figure 1).

**Figure 2:**
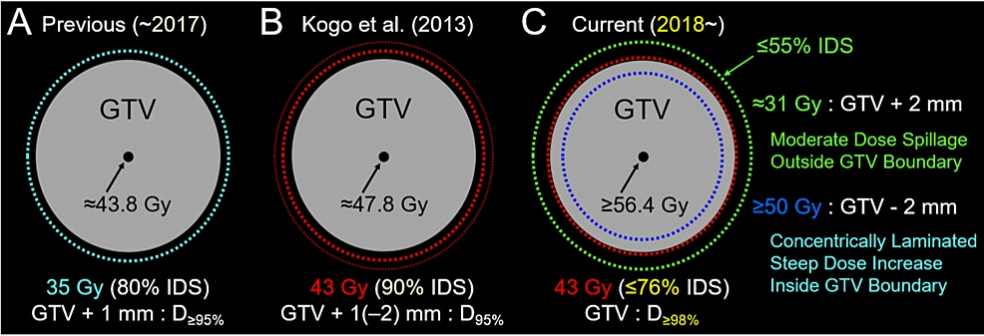
Difference in treatment planning for 5-fraction stereotactic radiosurgery The schemas show (A-C) a gross tumor volume (GTV) covered by the isodose surface (IDS) for dose prescription; our previous method until 2017 (A); the method reported by Kogo et al. in 2013 (B); and our current method since 2018 (C). (A-C) D_X%_ means a minimum dose covering ≥X% of the target volume. The percentage of the IDS is normalized to 100% at the maximum dose or isocenter dose. (A) In our previous protocol, the planning target volume (PTV) was generated by adding a uniform 1 mm margin to the GTV. The D_≥95%_ of the PTV was covered by 35 Gy with 80% IDS. (B) According to Kogo et al., the PTV was defined as the GTV + 1 mm, while 2 mm margin was added to the GTV for >15 cm^3^ and/or cystic tumors. The D_95%_ of the PTV was covered by 43 Gy with 90% IDS. (C) The GTV boundary is the base for dose prescription and planning. Compared to the method by Kogo et al., the normal tissue dose outside the GTV boundary is generally low, while the internal dose of the GTV is high.

An MRI revealed a tiny lesion in the right insular cortex (data not shown). These two lesions were treated simultaneously with 5-fr SRS using CyberKnife (CK) M6® (Sunnyvale, CA: Accuray Inc.) with 6 MV x-rays [4]. Our current treatment planning method for 5-fr SRS is described in Figure 2, along with the previous method and the method proposed by Kogo et al. [9].

**Figure 3:**
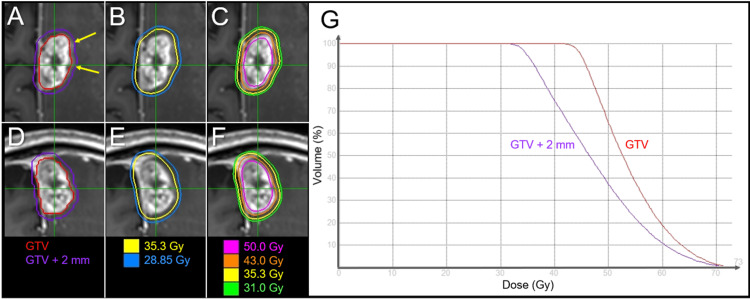
Target definition, dose distribution, and dose-volume histograms for five-fraction stereotactic radiosurgery The images show (A-F) CE-T1-WIs with target volumes (A, D) and dose distributions (B, C, E, F); axial views (A-C); coronal views (D-F); and dose-volume histograms (G). (A, D) Gross tumor volume (GTV) and 2-mm margin-added object volume (GTV + 2 mm). (A) Faint exudation (arrows in A) beyond the solid enhancing mass was excluded from the GTV, but included in the GTV + 2 mm volume. (D) In the coronal view, the contour line of the GTV is not smooth and jagged. CE: contrast-enhanced; WIs: weighted images

The optimization algorithm for SRS planning was CK-VOLO® (Sunnyvale, CA: Accuray Inc.), built into the dedicated planning system Precision® (Sunnyvale, CA: Accuray Inc.). Instead of separate planning for each tumor, simultaneous and comprehensive optimization with a single plan (path) was used to efficiently irradiate two lesions with 69 beams from 38 nodes, for which a regular dodecagon-shaped variable-sized collimator, Iris® (Sunnyvale, CA: Accuray Inc.), was selected with collimator sizes of 15, 20, 25, and 30 mm for a large BM and 7.5 and 10 mm for a tiny BM [4]. The GTV of the left frontal BM was defined as a solid enhancing lesion that was almost consistent with the visible mass on T2-weighted images (T1/T2 matching) [4,12,13]. The estimated treatment time was 23 minutes per fraction. The dose distribution and relevant planning parameters for a large BM are shown in Figure 3 and Table 1.

**Table 1:** Planning parameters of the gross tumor volume and relevant structures BED_10_: biological effective dose based on the linear-quadratic formula with an alpha/beta ratio of 10; GTV: gross tumor volume; D_max_: maximum dose; D_X%_: the minimum dose encompassing at least X% of the target volume; D_min_: minimum dose

Evaluation index	Dose	BED_10_
GTV D_max_ (D_0.001 cc_)	72.8 Gy	178.8 Gy
GTV D_2%_	68.9 Gy	163.8 Gy
GTV D_50%_	52.6 Gy	107.9 Gy
GTV – 2 mm D_98%_	50.3 Gy	100.9 Gy
GTV D_98%_	43.9 Gy	82.4 Gy
GTV D_99.2%_	43.0 Gy	80.0 Gy
GTV D_min_	40.8 Gy	74.1 Gy
GTV + 2 mm D_93.7%_	35.3 Gy	60.2 Gy
GTV + 2 mm D_94.5%_	35.0 Gy	59.5 Gy
GTV + 2 mm D_98%_	33.8 Gy	56.7 Gy
GTV + 2 mm D_99.9%_	31.0 Gy	50.2 Gy

**Figure 4:**
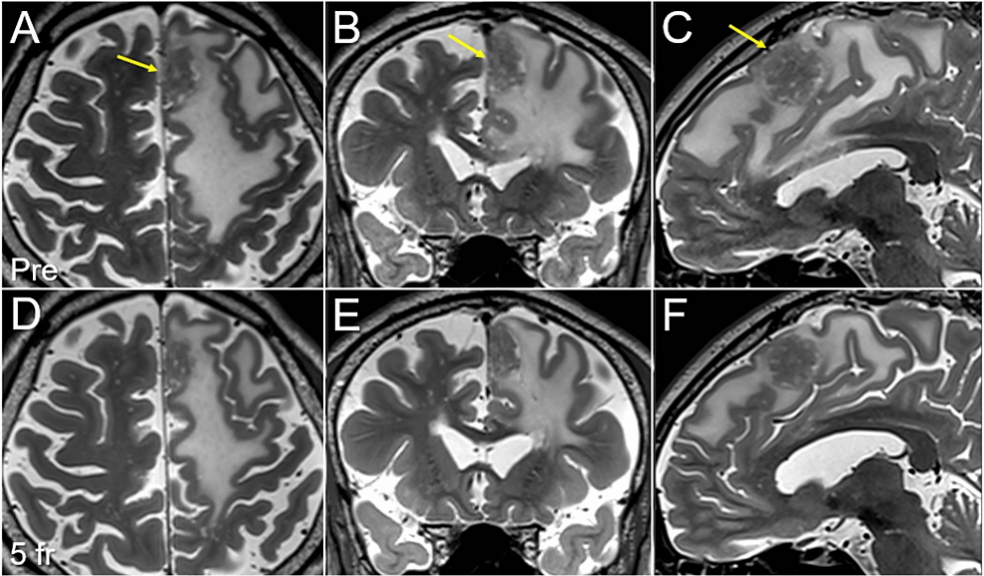
T2-weighted images before and at the completion of five-fraction stereotactic radiosurgery The images show axial images (A, D); coronal images (B, E); sagittal images (C, F); three days before initiation of SRS (A-C); and at the completion of five-fr SRS (seven days after initiation of SRS) (D-F). (A-C) The para-falcine mass lesion (arrows in A-C) is seen. (D-F) At the completion of SRS, shrinkage of the mass lesion along with mitigation of the perilesional edema and mass effect was observed. SRS: stereotactic radiosurgery; fr: fraction

The coverage values of the GTV with 43 Gy and the GTV + 2 mm structure with 35 and 31 Gy were 99.2%, 94.5%, and 99.9%, respectively. The irradiated isodose volume receiving ≥24 Gy, including the GTV (24 Gy volume), was 15.41 cm^3^ (<20 cm^3^) [6,7]. The surrounding tissue volume outside the GTV, receiving ≥28.85 Gy (V_28.85 Gy_) was 6.63 cm^3^ (<7 cm^3^) [14]. The physical dose of 28.85 Gy corresponds to a single dose of 14 Gy, according to the BED based on the LQ formula with an alpha/beta ratio of 2 (BED_2_) [14]. Meanwhile, the planning parameters for a tiny BM (0.05 cm^3^) were as follows: the minimum dose (D_min_) of 44.1 Gy, the mean dose of 46.8 Gy, the D_max_ of 48.6 Gy for the GTV, and the D_min_ of 32.6 Gy for the GTV + 2 mm. SRS was initiated on day three after the acquisition of the planning images [3,4,11,15]. Obvious tumor shrinkage, along with mitigation of the perilesional edema and mass effect, was observed on T2-WIs obtained at the completion of SRS (Figure 4).

**Figure 5:**
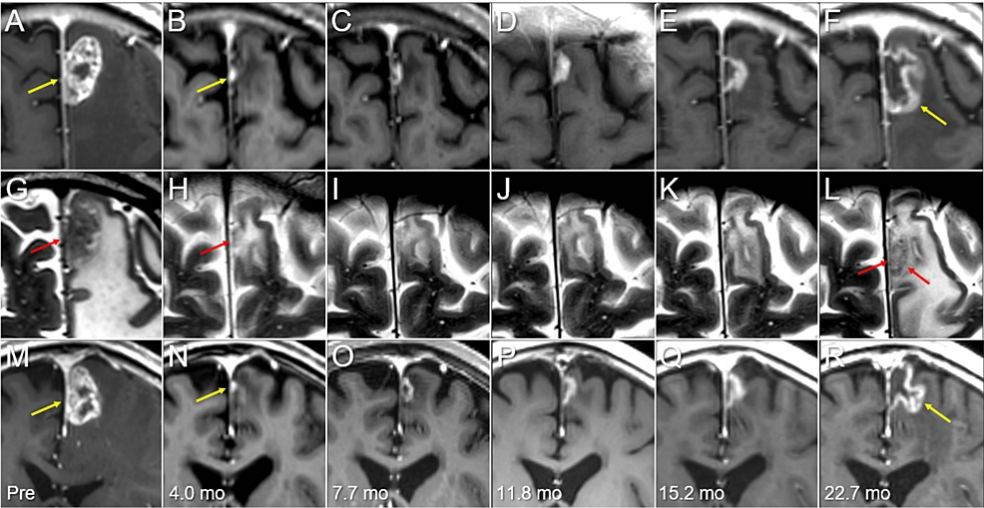
Magnetic resonance images before and after five-fraction stereotactic radiosurgery The images show axial CE-T1-WIs (A-F); axial T2-WIs (G-L); coronal CE-T1-WIs (M-R); 3 days before (pre) SRS (A, G, M); at four months (mo) after SRS (B, H, N); at 7.7 months (C, I, O); at 11.8 months (D, J, P); at 15.2 months (E, K, Q); and at 22.7 months (F, L, R). (A-R) These images are shown at the same magnification and coordinates under co-registration and fusion. (B, H, N) The BM (arrows in A, G, and M) showed remarkable shrinkage at four months, with the tumor remnant being seen as a tiny lesion (arrows in B, H, and N). (C-F, I-L, O-R) The tiny enhancing lesion gradually increased from 7.7 months to 22.7 months. Although no corresponding mass lesion was observed on T2-WI until 15.2 months, i.e., T1/T2 mismatch, the irregularly-shaped mass lesion (arrows in L) appeared at 22.7 months, which was still much smaller than the enhancing lesion (arrows in F, R) (T1/T2 mismatch). CE: contrast-enhanced; WI: weighted image; SRS: stereotactic radiosurgery; BM: brain metastasis

No significant displacement of the tumor relative to the cranium was observed [11,15]. These results indicate that the boundaries of the GTV and 2-mm outside the GTV received substantially higher doses than planned [4,14,15]. Neurological symptoms improved during irradiation and fully resolved within one month. Although a new lesion was detected in the right lower lobe of the lung, anti-cancer pharmacotherapy was refrained thereafter due to IPF. The tumor response after SRS for the large BM is shown in detail in Figure 5.

**Figure 6:**
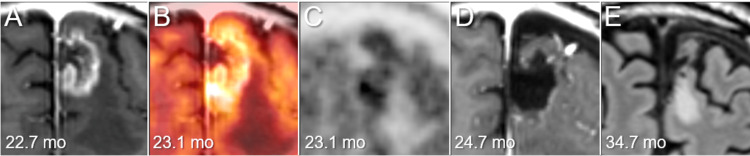
Axial images before and after the lesionectomy The images show CE-T1-WI at 22.7 months (mo) after SRS (A); a fusion image of CE-T1-WI at 22.7 months and an ^11^C-methionine (Met) positron emission tomography (PET) image at 23.1 months (B); ^11^C-Met-PET image at 23.1 months (C); CE-T1-WI at 24.7 months after SRS (three days after the lesionectomy) (D); and a fluid-attenuated inversion recovery image at 34.7 months after SRS (6.6 months after the lesionectomy) (E). (A-E) These images are shown at the same magnification and coordinates under co-registration and fusion. (B, C) ^11^C-Met-PET showed increased uptake in the enhancing lesion. CE: contrast-enhanced; WI: weighted image; SRS: stereotactic radiosurgery

Four months after SRS, nCR of the BMs was confirmed both clinically and radiographically. However, the tiny enhancing lesion in the left frontal lobe gradually enlarged from six months to two years. Given the relatively high dose prescribed to the tumor and the T1/T2 mismatch, tumor progression (≥20% and/or ≥5 mm enlargement following the nadir response of an enhancing lesion) was deemed to mainly reflect ARE [4,10,13]. However, because tumor recurrence could not be excluded regarding the progressive enlargement of the enhancing lesion, ^11^C-methionine (Met) positron emission tomography (PET) was acquired at 23.1 months, which showed increased uptake over a wide area of the enhancing lesion, suggesting the existence of a viable tumor (Figure 6) [16].

Therefore, minimally invasive salvage surgery was planned considering that the impaired pulmonary function was not amenable to general anesthesia, and a total lesionectomy was performed under local anesthesia [17]. Under neuronavigation guidance, a 4-cm linear skin incision followed by a 3-cm small craniotomy was made just above the lesion. After the corticotomy, the lesion was completely removed using neuroendoscopy [17]. The tumor invaded near the pia mater facing the falx; however, it did not invade the arachnoid membrane or dura mater. Thus, the lesion was deemed to be an intraparenchymal metastasis and not of meningeal-based extra-axial origin. The patient recovered uneventfully postoperatively. Pathological examination revealed viable tumor tissue mixed with necrosis. Postoperative MRI revealed a nearly complete removal of the lesion (Figure 6D).

Two doses of steroid pulse therapy were required for the AEs of IPF at two and four months after SRS. As the dyspnea on effort progressed, pirfenidone was changed to nintedanib five months after SRS. Nintedanib administration was discontinued 34 months after SRS [18]. No obvious tumor progression was observed on non-contrast MRI six months after lesionectomy (Figure 6E), while the tiny BM showed sustained nCR (data not shown). At the last follow-up visit, 41.1 months after SRS, the patient showed no apparent neurological deterioration relevant to intracranial metastases, without a significant decline in activities of daily living.

## Discussion

Since 2018, significant improvements in long-term sustained tumor regression and reduction of ARE have been observed in mfSRS in our facilities as a result of the limitation of targets, dose escalation, and extremely inhomogeneous target doses [3,4,10-12]. Many cases that appeared to be dominant in brain radionecrosis before 2017 were actually found to be predominantly smoldering tumors after a partial response. However, throughout the clinical course of the present case, we reaffirmed that pathological CR after SRS for BMs can be achieved by complete tumor necrosis, including possible microscopic brain infiltration up to 2-3 mm in depth, depending on the volume and histology [19]. Even if the radiographic maximal response after SRS is excellent if a slightly viable tumor remnant remains, regrowth will eventually occur unless controlled with anti-cancer pharmacotherapy. Maximum response with nCR or excellent tumor shrinkage within several months after SRS does not guarantee complete tumor necrosis. In BMs from lung adenocarcinomas harboring driver gene mutations, long-term CR can be achieved using relatively low-dose SRS and tyrosine kinase inhibitors [2]. CK® is one of the platforms with the highest accuracy among frameless SRS systems, as it enables frequent skull matching and correction during irradiation [4]. Despite the substantially high prescribed dose inside the GTV margin, the patient required salvage surgery for presymptomatic regrowth within two years. This result suggests an insufficient dose and/or coverage for achieving >1-2 year local control in the present BM from lung SCC. Although the tiny lesion showed sustained CR, a larger lesion would require a more sufficient dose and coverage [9,11]. Given the need for BED_10_ of ≥100 Gy for local control of the primary tumor of NSCLC, a higher dose of BED_10_ of ≥90 Gy may be necessary for obtaining better local control. In addition, the tumor mass was surrounded by a single layer of structures prior to SRS (Figure 1). The layered structure was initially considered to be the cerebral cortex compressed by extra-axial metastasis. However, the mass was confirmed intraoperatively as a parenchymal metastasis. Therefore, this layered structure may represent a reactive change, such as gliosis, within the white matter rather than in the cortex. In any case, it cannot be excluded that there was insufficient coverage of the medial microscopic brain invasion beyond the GTV + 2 mm boundary [19]. The clinical course of the present case reinforces the need for at least 43 Gy to the GTV margin to achieve a high probability of long-term local tumor control.

Despite progressive enlargement of the enhancing lesion, T2-WI did not reveal any visible mass lesions until just prior to salvage surgery in the present case. This showed that the T1/T2 mismatch was not always reliable in excluding the possibility of recurrence [10,13]. In contrast, the increased uptake of ^11^C-Met-PET was useful for estimating pathology [16]. Anti-cancer medication was not consistently administered in the present case because of IPF. However, since nintedanib has anti-angiogenic effects in addition to its anti-fibrotic effects, post-SRS administration of nintedanib for IPF might provide some anti-tumor efficacy for lung cancer and mitigate ARE relevant to SRS [18].

In mfSRS, substantial variability remains regarding the target definition (PTV margin), dose/fractionation, BED definition, and target dose heterogeneity [5,11,20]. Accordingly, there is a need to identify methods that can achieve long-term sustained tumor shrinkage and safety, along with excellent short-term tumor response. The treatment and clinical course in the present case will contribute to the identification of an optimal method for achieving long-term efficacy and safety among the various currently implemented mfSRSs.

## Conclusions

A lung SCC case of large symptomatic BM was presented, which was treated with five-fr SRS using the BED10 of ≥80 Gy to the GTV boundary with an internal steep dose increase. Despite obvious tumor shrinkage during irradiation, the BM demonstrated gradual tumor regrowth following the nadir response, with nearly complete remission. The present case suggests that even ≥43 Gy with ≤60% isodose to the GTV margin and ≥31-35 Gy to the 2 mm outside the GTV boundary are insufficient to achieve long-term local tumor control by five-fr SRS alone in some large BM from lung SCC. A more sufficient dose and/or coverage is required to achieve complete tumor necrosis, including potential microscopic brain invasion.
